# Complete Genome and Plasmid Sequences for *Rhodococcus fascians* D188 and Draft Sequences for *Rhodococcus* Isolates PBTS 1 and PBTS 2

**DOI:** 10.1128/genomeA.00495-16

**Published:** 2016-06-09

**Authors:** Rio A. Stamler, Danny Vereecke, Yucheng Zhang, Faye Schilkey, Nico Devitt, Jennifer J. Randall

**Affiliations:** a Department of Entomology, Plant Pathology and Weed Science, New Mexico State University, Las Cruces, New Mexico, USA; b Department of Applied Biosciences, Ghent University, Ghent, Belgium; c Department of Plant Pathology, University of Florida, Gainesville, Florida, USA; d National Center for Genome Resources, Santa Fe, New Mexico, USA

## Abstract

*Rhodococcus fascians*, a phytopathogen that alters plant development, inflicts significant losses in plant production around the world. We report here the complete genome sequence of *R. fascians* D188, a well-characterized model isolate, and *Rhodococcus* species PBTS (pistachio bushy top syndrome) 1 and 2, which were shown to be responsible for a disease outbreak in pistachios.

## GENOME ANNOUNCEMENT

*Rhodococcus fascians*, the sole characterized plant pathogen in the genus *Rhodococcus*, is a Gram-positive pleomorphic bacterium that causes disease through the production and modulation of plant growth regulators, such as cytokinins and auxins (1). *R. fascians* infects a large variety of plants, including monocots and dicots, often causing cryptic symptomology (2). *R. fascians* D188, which has intensively been studied for decades, has revealed a wealth of knowledge about this plant pathogen, including chromosomal and plasmid localized virulence genes necessary for symptomatic host colonization and cytokinin production (3, 4). The virulence plasmid pFiD188 was previously sequenced using Sanger technology, and fragmented draft genome sequences for multiple *R. fascians* isolates, including D188, are available (5, 6). Pistachio bushy top syndrome (PBTS) describes a suite of symptoms affecting clonally propagated UCB-1, an interspecific hybrid rootstock planted in California, Arizona, and New Mexico (7, 8). Two distinct *Rhodococcus* species isolates (PBTS 1 and PBTS 2) were isolated from diseased trees and shown to cause stunting and abnormal root and shoot development in pathogenicity assays. Given the global scale of nursery plant production and the diverse population structure of *R. fascians*, as indicated by initial genome sequences (6), high-quality complete reference sequences are needed to fully characterize newly discovered isolates. The reference *R. fascians* strain D188 (from the private collection of D. Vereecke) and PBTS isolates (7) were stored in glycerol stocks at -80°C. Cultures were grown in 50 ml of LB broth, and DNA was extracted using a Wizard genomic DNA purification kit, according to the manufacturer’s instructions (Promega, Madison, WI). DNA was shipped on dry ice to the National Center for Genomic Resources (NCGR, Santa Fe, NM) for single-molecule real-time (SMRT) sequencing. Libraries with a 10-kb insert were prepared, and each isolate was sequenced using one SMRT cell on the PacBio RS II instrument (Pacific Biosciences, Menlo Park, CA). Hierarchical Genome Assembly Process (HGAP) was used for *de novo* assembly (9) (RS_HGAP assembly.2; Pacific Biosciences). For strain D188, an additional round of polishing was performed using only reads with a quality of ≥84. The genomes were annotated with Prokka version 1.12-beta (10), protein-coding features were predicted using Prodigal version 2.6 (11), tRNA was predicted by ARAGORN version 1.2 (12), and rRNA was predicted by RNAmmer version 1.2 (13). The genome annotations reveal a wealth of predicted secondary metabolism-related genes, including novel antibiotic synthesis, heavy metal resistance, siderophore production, pilus-like assembly mechanisms, secreted proteins, transposons and other mobile elements, and prophage sequences.

### Nucleotide sequence accession numbers.

The complete genome and plasmid sequences for this whole-genome shotgun project have been deposited at DDBJ/EMBL/GenBank under the following accession numbers: CP015235, CP015236, and CP015237 for D188, CP015219 for PBTS 1, and CP015220 and CP015221 for PBTS 2. The versions described in this paper are CP015235.1, CP015236.1, CP015237.1, CP015219.1, CP015220.1, and CP015221.1, respectively.

## References

[1] Stes E, Vandeputte OM, El Jaziri M, Holsters M, Vereecke D 2011 A successful bacterial coup d’état: how *Rhodococcus fascians* redirects plant development. Annu Rev Phytopathol 49:69–86. doi:10.1146/annurev-phyto-072910-095217. 21495844

[2] Putnam ML, Miller ML 2007 *Rhodococcus fascians* in herbaceous perennials. Plant Dis 91:1064–1076. doi:10.1094/PDIS-91-9-1064. 30780643

[3] Vereecke D, Cornelis K, Temmerman W, Jaziri M, Van Montagu M, Holsters M, Goethals K 2002 Chromosomal locus that affects pathogenicity of *Rhodococcus fascians*. J Bacteriol 184:1112–1120. doi:10.1128/jb.184.4.1112-1120.2002. 11807072 PMC134788

[4] Crespi M, Vereecke D, Temmerman W, Van Montagu M, Desomer J 1994 The *fas* operon of *Rhodococcus fascians* encodes new genes required for efficient fasciation of host plants. J Bacteriol 176:2492–2501. 8169198 10.1128/jb.176.9.2492-2501.1994 PMC205384

[5] Francis I, De Keyser A, De Backer P, Simón-Mateo C, Kalkus J, Pertry I, Ardiles-Diaz W, De Rycke R, Vandeputte OM, El Jaziri M, Holsters M, Vereecke D 2012 pFiD188, the linear virulence plasmid of *Rhodococcus fascians* D188. Mol Plant Microbe Interact 25:637–647. doi:10.1094/MPMI-08-11-0215. 22482837

[6] Creason AL, Vandeputte OM, Savory EA, Davis EW II, Putnam ML, Hu E, Swader-Hines D, Mol A, Baucher M, Prinsen E, Zdanowska M, Givan SA, El Jaziri M, Loper JE, Mahmud T, Chang JH 2014 Analysis of genome sequences from plant pathogenic *Rhodococcus* reveals genetic novelties in virulence loci. PLoS One 9:e101996. doi:10.1371/journal.pone.0101996. 25010934 PMC4092121

[7] Stamler RA, Kilcrease J, Fichtner E, Kallsen C, Cooke P, Heerema RJ, Randall JJ 2015 First report of *Rhodococcus* isolates causing pistachio bushy top syndrome on ‘UCB-1’ rootstock in California and Arizona. Plant Dis 99:1468–1476. doi:10.1094/PDIS-12-14-1340-RE. 30695969

[8] Stamler RA, Heerema R, Randall JJ 2015 First report of phytopathogenic *Rhodococcus* isolates on pistachio bushy top syndrome ‘UCB-1’ rootstock in New Mexico. Plant Dis 99:1854. doi:10.1094/PDIS-04-15-0471-PDN. 30695969

[9] Chin CS, Alexander DH, Marks P, Klammer AA, Drake J, Heiner C, Clum A, Copeland A, Huddleston J, Eichler EE, Turner SW, Korlach J 2013 Nonhybrid, finished microbial genome assemblies from long-read SMRT sequencing data. Nat Methods 10:563–569. doi:10.1038/nmeth.2474. 23644548

[10] Seemann T 2014 Prokka: rapid prokaryotic genome annotation. Bioinformatics 30:2068–2069. doi:10.1093/bioinformatics/btu153. 24642063

[11] Hyatt D, Chen GL, LoCascio PF, Land ML, Larimer FW, Hauser LJ 2010 Prodigal: prokaryotic gene recognition and translation initiation site identification. BMC Bioinformatics 11:119. doi:10.1186/1471-2105-11-119. 20211023 PMC2848648

[12] Laslett D, Canback B 2004 ARAGORN, a program to detect tRNA genes and tmRNA genes in nucleotide sequences. Nucleic Acids Res 32:11–16. doi:10.1093/nar/gkh152. 14704338 PMC373265

[13] Lagesen K, Hallin P, Rødland EA, Staerfeldt H-H, Rognes T, Ussery DW 2007 RNAmmer: consistent and rapid annotation of ribosomal RNA genes. Nucleic Acids Res 35:3100–3108. doi:10.1093/nar/gkm160. 17452365 PMC1888812

